# Involvement of patients or their representatives in quality management functions in EU hospitals: implementation and impact on patient-centred care strategies

**DOI:** 10.1093/intqhc/mzu022

**Published:** 2014-03-09

**Authors:** Oliver Groene, Rosa Sunol, Niek S. Klazinga, Aolin Wang, Maral Dersarkissian, Caroline A. Thompson, Andrew Thompson, Onyebuchi A. Arah, on behalf of the DUQuE Project Consortium, N Klazinga, DS Kringos, MJMH Lombarts, T Plochg, MA Lopez, M Secanell, R Sunol, P Vallejo, P Bartels, S Kristensen, P Michel, F Saillour-Glenisson, F Vlcek, M Car, S Jones, E Klaus, S Bottaro, P Garel, M Saluvan, C Bruneau, A Depaigne-Loth, C Shaw, A Hammer, O Ommen, H Pfaff, O Groene, D Botje, C Wagner, H Kutaj-Wasikowska, B Kutryba, A Escoval, A Lívio, M Eiras, M Franca, I Leite, F Almeman, H Kus, K Ozturk, R Mannion, OA Arah, M DerSarkissian, CA Thompson, A Wang, A Thompson

**Affiliations:** 1 Department of Health Services Research and Policy, London School of Hygiene & Tropical Medicine, London, UK; 2 Avedis Donabedian Research Institute (FAD), Universitat Autonoma de Barcelona, Barcelona, Spain; 3 Red de Investigación en Servicios de Salud en Enfermedades Crónicas (REDISSEC), Barcelona, Spain; 4 Department of Public Health, Academic Medical Center, University of Amsterdam, Amsterdam, The Netherlands; 5 Department of Epidemiology, Fielding School of Public Health, University of California, Los Angeles (UCLA), Los Angeles, CA, USA; 6 Palo Alto Medical Foundation Research Institute, Palo Alto, CA, USA; 7 School of Social and Political Science, University of Edinburgh, Edinburgh, UK; 8 UCLA Center for Health Policy Research, Los Angeles, CA, USA

**Keywords:** quality management, quality measurement, patient-centred care, hospital care, hospital, patient involvement

## Abstract

**Objective:**

The objective of this study was to describe the involvement of patients or their representatives in quality management (QM) functions and to assess associations between levels of involvement and the implementation of patient-centred care strategies.

**Design:**

A cross-sectional, multilevel study design that surveyed quality managers and department heads and data from an organizational audit.

**Setting:**

Randomly selected hospitals (*n* = 74) from seven European countries (The Czech Republic, France, Germany, Poland, Portugal, Spain and Turkey).

**Participants:**

Hospital quality managers (*n* = 74) and heads of clinical departments (*n* = 262) in charge of four patient pathways (acute myocardial infarction, stroke, hip fracture and deliveries) participated in the data collection between May 2011 and February 2012.

**Main Outcome Measures:**

Four items reflecting essential patient-centred care strategies based on an on-site hospital visit: (1) formal survey seeking views of patients and carers, (2) written policies on patients' rights, (3) patient information literature including guidelines and (4) fact sheets for post-discharge care. The main predictors were patient involvement in QM at the (i) hospital level and (ii) pathway level.

**Results:**

Current levels of involving patients and their representatives in QM functions in European hospitals are low at hospital level (mean score 1.6 on a scale of 0 to 5, SD 0.7), but even lower at departmental level (mean 0.6, SD 0.7). We did not detect associations between levels of involving patients and their representatives in QM functions and the implementation of patient-centred care strategies; however, the smallest hospitals were more likely to have implemented patient-centred care strategies.

**Conclusions:**

There is insufficient evidence that involving patients and their representatives in QM leads to establishing or implementing strategies and procedures that facilitate patient-centred care; however, lack of evidence should not be interpreted as evidence of no effect.

## Background

The role of involving patients in designing and assessing health care services has increased substantially over the last few years [1]. Approaches towards user involvement go back over decades, motivated mainly by ethical and governance concerns [2]. More recently, the patient safety movement has demonstrated the need to involve patients and learn from their experiences in order to ensure safe and high quality patient-centred care [3, 4]. The involvement of patients in designing and assessing their care is seen as a strategy to promote patient-centred care, acknowledged as an integral dimension of health care quality [4, 5]. This is highlighted in various recommendations of the high-profile inquiry into the quality and safety concerns at Mid-Staffordshire Foundation NHS Trust that specifically emphasizes the importance of patient representation and the prioritizing of patients' needs in the organization and delivery of health care [6].

Patient involvement is referred to under different terms in the literature, including ‘patient and public involvement’, ‘user involvement’, ‘lay involvement’ or ‘patient representation’ [7]. We will focus here on the established term patient involvement to denote the involvement of patients or their representatives in activities related to planning, designing or assessing quality management (QM) in hospitals. The involvement of patients and their representatives in health care takes many forms, ranging from lay membership in managerial boards to involvement in condition-specific activities, such as services design, development of patient information material, identification of improvement priorities, or assessing and interpreting results of patient surveys. Hospitals recognized for their leadership in the field of patient-centred care involve patients or patient representatives in formal quality functions (such as setting standards or targets, and discussing results).

Yet, the evidence base on the involvement of patients and patient representatives in quality functions is limited. For example, it has been demonstrated that patient involvement is frequently limited to the highest organizational level and not devolved to clinical units [8]. Moreover, it focuses mainly on the least technical issues of care (e.g. patient education) where patients can make constructive contributions; however, these contributions are not necessarily acted on subsequently [9]. Other studies exhibited that patient involvement is often professionally led and can be resource intensive and its gains are hard to identify [10]. One of the few studies to explore the relationship between hospital organizational characteristics and consumer involvement quantitatively detected only a low and non-significant correlation [11].

Given the dearth of research on the actual involvement of patients or their representatives in formal QM roles [1, 12] and the effect of such strategies on organizational policies and procedures [13], the aims of this study were (i) to describe the involvement of patients or their representatives in QM at hospital and at department (or pathway) levels and (ii) to investigate the effect of involvement on the implementation of strategies to improve patient-centredness of care in European hospitals.

## Methods

### Study design, setting and population

This study was conducted as part of the ‘Deepening our understanding of quality improvement in Europe (DUQuE)’ project, which was funded by the EU 7th Research Framework Program [14]. The overall aims and objectives of the project are described in detail elsewhere [15]. We employed a cross-sectional, multilevel study design in which patient-level measurements are nested in hospital departments, which are in turn nested in hospitals in seven EU countries. Hospitals were randomly selected from the participating countries: Czech Republic, France, Germany, Poland, Portugal, Spain and Turkey between May 2011 and January 2012.

Each hospital nominated a coordinator (usually the quality manager) who attended training sessions on sampling procedures and data collection. Data collection included questionnaires to professionals and patients, chart review, administrative data and an organizational audit. Full details of the field test are described elsewhere [15]. The data reported in this paper emanates from surveys to the hospital quality manager, heads of departments and an on-site audit on the implementation of patient-centred care strategies. For the audit, training sessions were carried out with independent surveyors in each country. The selection criteria for these surveyors differed slightly between country, but a general requirement was independence of the hospital setting undergoing audit and experience in accreditation visits to assess hospital quality and safety. An IT platform was created to collect data from electronic surveys and the organizational audit. Responses rates were high (89% for professional questionnaires and 99% for the organizational audit).

### Conceptual framework

**Figure 1:**
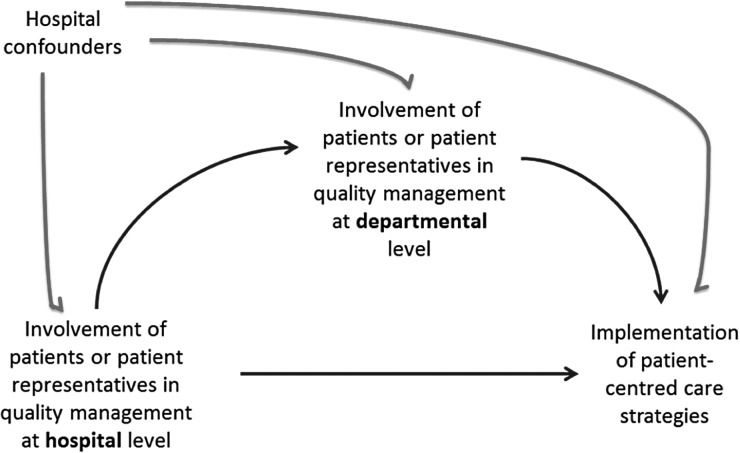
Directed acyclic graph for the relationships between patient involvement in QM at hospital and departmental levels and patient-centred care strategies.

We used a directed acyclic graph (DAG) to depict our knowledge and assumptions about the relationships under study and to guide our covariate selection for confounding control in the statistical analysis [16]. Although this study has a cross-sectional design, it is still useful to make the assumed and hypothesized relationships transparent with the visual aid of a DAG. The DAG in Fig. 1 reflects how patient involvement in QM at hospital and departmental levels can influence patient-centred care strategies. Hospital confounders (size, ownership and teaching status) potentially affect each of these measures.

### Outcomes, predictors and covariates

The outcome variable is a score reflecting the implementation of essential patient-centred care strategies at the departmental level, measured through on-site visit for each of the four departments. This construct contains the following items: (a) a formal survey seeking views of patients and carers, (b) written policies on patients' rights, (c) patient information literature including guidelines and (d) fact sheets for post-discharge care. Each item is scored on a five-point scale (no or negligible compliance, low compliance, medium compliance, high-extensive compliance and full compliance), with further instructions to account for the extent of implementation such as whether patient surveys had been fully documented and reported, whether patient education material had been designed in a non-technical language or whether evidence indicated an organization-wide implementation. The outcome measure was assessed by independent hospital surveyors during an on-site hospital visit. This visit lasted on average 1.5 days and covered overall hospital organization and organizational procedures implemented in the four pathways reported here. This outcome measure was developed based on a review of accreditation and quality standards, policy documents and the literature and builds on previous research [17]. We acknowledge that patient-centeredness is a broader construct than reflected by these four items, as discussed in our own research [18, 19]. However, in the context of this study, our primary interest was to assess the implementation of patient-centred care strategies as stipulated in relevant policies on hospital QM systems. These aspects are most frequently covered by the chosen items.

The key predictor variable is the score on the involvement of patients and their representatives in QM functions. This was measured for each hospital once at the hospital level and then again for each of the four departments. These constructs are based on a five-item scale that has been validated and used in previous research [17]. Items assess whether patients are involved in (i) the development of quality criteria/standards/protocols, (ii) the design/organization of processes, (iii) quality committees, (iv) quality improvement projects and (v) discussion of results of quality improvement projects. Each item is scored on a four-point scale including the categories never, sometimes, usually and always. The mean score of these four items is used in the associational analysis. Covariates include country and hospital level (size of the hospital, ownership and teaching status).

### Analysis

The main hypothesis is that the involvement of patients and their representatives at hospital and/or departmental level is positively associated with essential patient-centred care strategies. This is based on the assumption that involving patients in designing and assessing care would lead to implementation of policies to support patient views, rights and access to relevant patient information. We first present descriptive statistics on characteristics of hospitals participating in the study, and department-specific means and SDs for the main predictors and outcomes in our study. We also describe items that were aggregated to build the hospital- and department-level patient involvement and patient information constructs. Fisher's exact test or Pearson's χ^2^ statistics were used to determine whether there were significant differences in these items across pathways, as appropriate.

Covariate adjustments in our multilevel models were guided by the DAG shown in Fig. 1, which we assumed to be representative of the data-generating mechanism. These models were restricted to complete observations for the predictor, outcome and all confounders in the analysis. Two separate sets of models were used to investigate (i) whether patient involvement in QM at the ‘hospital’ level was associated with patient-centred care strategies at the ‘department’ level and (ii) whether patient involvement at the ‘department’ level was associated with patient-centred care strategies.

We used multivariate linear mixed models with a random intercept by country to account for clustering of hospitals within countries and adjusted for hospital size, ownership and teaching status. Models assessing the association between department-level patient involvement and patient-centred care strategies were further adjusted for hospital-level patient involvement, as this variable is a confounder in our DAG. All statistical analyses were carried out in SAS (version 9.3, SAS Institute Inc., NC, USA, 2011).

## Results

Overall, 74 hospitals contributed data to be included in this study. The majority were public hospitals (79.7%) and about half (44.5%) were teaching hospitals. Larger hospitals with >500 beds accounted for more than half of the hospitals in the sample. A total of 72 of the 74 hospitals completed the hospital quality manager and head of department questionnaires (Table 1).

**Table 1:** Characteristics of hospitals participating in study

Characteristic	*N*	%
All Hospitals	74	100
Czech Republic	12	16.2
France	11	14.8
Germany	4	5.4
Poland	12	16.2
Portugal	11	14.8
Spain	12	16.2
Turkey	12	16.2
Teaching hospitals	33	44.5
Public hospitals	59	79.7
Approximate number of beds in hospital		
<200	7	9.4
200–500	22	29.7
501–1000	31	41.8
>1000	14	18.9
Completed hospital quality manager questionnaires	72	97.3
Competed head of department questionnaires	72	97.3

In Table 2, we provide descriptive statistics for predictor and outcome variables. The score for the predictor variable is much higher at hospital level, whereas scores for the involvement of patients and their representatives are universally lower across departments. The score of the outcome variable is highest for deliveries and lowest for hip fracture care.

**Table 2:** Descriptive statistics for patient involvement at hospital and departmental levels and patient-centred care strategies

	All conditions	AMI	Deliveries	Hip fracture	Stroke
	Mean (SD)	Mean (SD)	Mean (SD)	Mean (SD)	Mean (SD)
Predictors					
Patient involvement in QM at ‘hospital' level (0–5)	1.6 (0.7)				
Patient involvement in QM at ‘departmental’ level (0–5)	0.6 (0.7)	0.8 (0.7)	0.6 (0.8)	0.6 (0.6)	0.6 (0.7)
Outcome					
Patient-centred care strategies (0–4)	2.8 (0.9)	2.8 (0.9)	3.0 (0.9)	2.6 (0.9)	2.8 (0.9)

Details of the implementation of strategies to involve patients and their representatives in QM functions are presented in Table A1. Patients and their representatives are rarely involved in developing quality criteria, designing/organizing processes of care, participating in quality committees or discussing results of quality improvement projects. Somewhat more frequently, they are involved in the implementation of quality improvement projects. There was little variation in the observed involvement across departments. As shown in Table A2, slightly more than half of the departments fully complied with having established formal surveys seeking the views of patients or carers and almost three-quarters had written policies on patients' rights in place. Roughly 38 and 43%, respectively, were fully compliant with providing patient information literature and fact sheets for post-discharge care. The implementation of these strategies differed across pathways for the latter two items, with child delivery care yielding higher responses for patient information (*P* = 0.054) and AMI care yielding higher responses for the implementation of post-discharge care (*P* = 0.033).

Results from our multilevel models show that there is insufficient evidence of an association between the involvement of patients and their representatives in QM functions at hospital level and the implementation of patient-centred care strategies in any of the four departments. We did not detect a relationship between patient-centred care strategies and type of hospital or hospital size (Table 3).

**Table 3:** Results of multivariable analysis for relationship of patient involvement in QM at the hospital level and patient-centred care strategies^a^

	Patient-centred care strategies											
	AMI (*N* = 69)			Deliveries (*N* = 69)			Hip Fracture (*N* = 71)			Stroke (*N* = 71)		
	*b*	SE	pr>|t|	*b*	SE	pr >|t|	*b*	SE	pr >|t|	*b*	SE	pr >|t|
Patients involved in QM at hospital level	0.19	0.15	0.211	0.06	0.14	0.667	0.04	0.15	0.784	0.17	0.15	0.253
Teaching hospital												
Yes	−0.08	0.29	0.793	−0.05	0.28	0.847	0.00	0.28	0.996	0.26	0.28	0.354
No	(reference)			(reference)			(reference)			(reference)		
Public hospital												
Yes	0.19	0.31	0.539	0.08	0.29	0.779	0.11	0.29	0.696	0.13	0.29	0.643
No	(reference)			(reference)			(reference)			(reference)		
Hospital size												
<200	(reference)			(reference)			(reference)			(reference)		
200–500	−0.38	0.41	0.359	−0.69	0.42	0.106	−0.20	0.41	0.631	−0.63	0.41	0.127
501–1000	0.10	0.42	0.808	−0.26	0.43	0.550	0.00	0.42	0.995	−0.23	0.41	0.583
>1000	0.15	0.47	0.757	−0.08	0.48	0.876	−0.39	0.47	0.415	−0.32	0.47	0.502

^a^Multivariate linear mixed models with a random intercept by country to account for clustering of hospitals within countries and adjusted for hospital size, ownership and teaching status.

A similar analysis was conducted to assess the relationship between departmental-level strategies for the involvement of patients and their representatives in QM function and the implementation of patient-centred care strategies. Again, our evidence did not support a relationship between either patient involvement or hospital-type and patient-centred care strategies in any of the pathways. However, we found that the smallest hospitals were more likely to implement patient-centred care strategies than larger hospitals (Table 4). This effect was observed for all pathways, with the exception of stroke care.

**Table 4:** Results of multivariate analysis of the relationship between patient involvement in QM at the department level and patient-centred care strategies^a^

	Patient-centred care strategies											
	AMI (*N* = 48)			Deliveries (*N* = 58)			Hip fracture (*N* = 57)			Stroke (*N* = 64)		
	*b*	SE	pr >|t|	*b*	SE	pr >|t|	*b*	SE	pr >|t|	*b*	SE	pr >|t|
Patients involved in QM at departmental level	0.29	0.18	0.112	0.03	0.13	0.833	0.21	0.19	0.277	−0.14	0.18	0.452
Patients involved in QM at hospital level	0.15	0.14	0.299	0.12	0.15	0.427	0.02	0.16	0.913	0.18	0.15	0.250
Teaching hospital												
Yes	−0.36	0.32	0.267	0.09	0.23	0.706	−0.22	0.33	0.524	0.15	0.30	0.630
No	(reference)			(reference)			(reference)			(reference)		
Public hospital												
Yes	0.15	0.33	0.646	−0.01	0.27	0.972	0.03	0.30	0.922	0.30	0.33	0.367
No	(reference)			(reference)			(reference)			(reference)		
Hospital size												
<200	(reference)			(reference)			(reference)			(reference)		
200–500	−1.77	0.52	0.002	−0.82	0.38	0.038	−1.25	0.61	0.047	−0.48	0.47	0.320
501–1000	−1.16	0.52	0.031	−0.38	0.37	0.317	−1.12	0.62	0.081	−0.06	0.48	0.900
>1000	−1.05	0.55	0.062	0.05	0.43	0.910	−1.30	0.64	0.049	−0.15	0.52	0.781

^a^Multivariate linear mixed models with a random intercept by country to account for clustering of hospitals within countries and adjusted for hospital size, ownership, teaching status, and ‘Patients involved in QM at hospital level’.

## Discussion

We aimed to describe the involvement of patients in QM at hospital and at department level and to investigate the effect of levels of involvement on organizational policies and procedures to improve patient-centred care strategies in European hospitals.

### Levels of implementation of patient involvement and patient-centred care strategies

Despite years of advocacy and policy support, the actual involvement of patients and their representatives in QM functions remains low in our study, in particular with regard to important domains such as establishing quality standards or organizing processes, for which evidence suggests that patients can make important contributions [20, 21]. The only domain where patients and their representatives appear to be involved to some extent is regarding the implementation of quality improvement projects. This finding is consistent and observed both at hospital and departmental level. At departmental level, patient involvement is less frequent than at hospital level, and there is little variation across patient pathways. This is supported by comparable studies suggesting that ‘beyond board level, involvement of users, patients […] and the general public is patchy and superficial.’ [8]. Given that the ratings on the level of patient involvement are based on self-reports by managers; the actual level of involvement may be even lower in practice. Patient-centred care strategies are implemented somewhat more heterogeneously, with pathways for deliveries yielding higher scores, although these differences do not reach statistical significance. The observed levels of implementation of key strategies to elicit and understand patient views and provide crucial information to patients are low. Among the four assessed pathways, the deliveries and acute myocardial infarction pathways report higher levels of implementation of patient-centred care strategies.

### Associations between patient involvement and patient-centred care strategies

We hypothesized that higher involvement of patient and patient representative involvement would be associated with efforts to understand patient's views by conducting formal surveys, establishing written policies on patient's rights, producing and providing patient information literature on the care pathway, and providing facts sheets for post-discharge care.

We were not able to detect any significant association between levels of patient involvement and implementation of patient-centred care strategies. This might be interpreted in various ways. First, it is possible that we did not detect an association because hospitals had involved patients and their representatives only recently and sufficient time has not passed for their involvement to lead to establishing or implementing policies or procedures. Secondly, patient involvement may have some positive and measurable effects; however, they do not affect the policies and procedures investigated here. For example, Mockford *et al*. demonstrated that patient and public involvement has a range of impacts on health care services, in particular around information development and dissemination, including patient information brochures [1]. However, previous research studies have not directly assessed the relationship we examined in this study. Thirdly, involving patients and their representatives in QM functions might have no measurable effect on the policies and procedures investigated here. It is conceivable that some hospitals strive for patient involvement without providing the resources or commitment to translate this into changes in service organization or delivery. In individual cases, a hospital might perform ‘lip service’, rather than a service transformation function, as reported elsewhere [9]. This might be a particular issue if patients and their representatives are not properly selected or mentored to make a contribution. Recent research confirms this hypothesis and suggests that for patient involvement to be effective, facilitators who can ‘support, engage, navigate and advocate’ for participation and influence are needed [22]. While examples from leading advocates of patient-centred care, such as the Dana Faber Institute and the Children's Hospital in Boston in the USA, provide evidence that patients make valuable contributions to QM work, the success of patient involvement may depend largely on being able to recruit patients with the right experience and their ability to express their views constructively [23]. The only effect observed in multivariate analysis was one of hospital size, where we detected an inverse relationship between levels of involvement of patients and their representatives in QM and the implementation of patient-centred care strategies. This might be explained by smaller hospitals being more community oriented than large teaching hospitals [24]. However, we did not detect a clear size gradient; thus, there might be other factors in the group of the smallest hospitals that explain higher implementation.

### Limitations

Our study has a number of limitations. Some of the common limitations of the DUQuE study are referred to elsewhere [15]. Specific limitations of this study are the lack to control for temporality, i.e. the duration or history of involving patients or their representatives in QM functions, as it might be assumed that it takes some time from first involving patients and their representatives to establishing relevant policies and procedures. An added complexity is that seven countries participating in the study might be at different stages on their journey to patient involvement. Ideally, it would have been desirable to highlight these rather than to adjust for the country effect in the multivariable analysis; however, the study was not designed to allow this. Another limitation is that we were not able to use information collected from hospital board members on the criteria for selection and training of patients and their representatives. This would have allowed illumination of the impact of these criteria on levels of involvement and strength of association with patient-centred care strategies. However, as described by Secanell *et al*., the response rate to this particular questionnaire was poor [15]. The involvement of patients itself in designing this research would have been very desirable, ideally as a mixed-methods study, however, this was infeasible given the complexity of the project, timescales, multiple settings in multiple countries and resources available [18]. Another limitation is that we were not able to assess the factors that motivated hospitals to involve patients and their representatives in QM functions. Knowledge of these factors might inform further research on the impact of involving patients and their representatives in hospital QM functions. A final limitation is that in this study we were not able to assess the ability of the involved patients to exert influence on the decision-making process regarding the organization of hospital services. This is an issue that has been addressed in previous studies, suggesting that levels of patient involvement that could be characterized as tokenism might be measurable with the scales used here but would not reflect the professionally led agenda for change and thus confound the relationship between involvement and outcomes [9, 10, 22, 25]. Given these limitations, the failure to detect statistically significant associations between patient involvement and the implementation of strategies to improve patient-centeredness of care should not deflect from the general benefits of patient involvement [12] and should not be interpreted as evidence of no relationship.

## Conclusion

Current levels of involving patients and their representatives in QM functions in European hospitals are low overall. It is slightly higher regarding hospital-wide activities as compared to departmental-specific functions and more frequent regarding specific projects than broader functions of planning and assessing results. We did not detect a relationship between levels of involving patients and their representatives in QM functions and the implementation of patient-centred care strategies. Further research should address the motivations and contextual factors for patient involvement in QM in greater detail. Currently, there is insufficient evidence to conclude that involving patients and their representatives lead to establishing or implementing policies and procedures that facilitate patient-centred care; however, this lack of evidence should not be interpreted as evidence of no effect.

## Funding

The study, “Deepening our Understanding of Quality Improvement in Europe (DUQuE)” has received funding from the European Community's Seventh Framework Programme (FP7/2007-2013) under grant agreement no 241822. Funding to pay the Open Access publication charges for this article was provided by European Community's Seventh Framework Programme (FP7/2007–2013) under grant agreement no 241822.

## Appendix 1

### The DUQuE Project Consortium comprises

Klazinga N, Kringos DS, Lombarts MJMH and Plochg T (Academic Medical Centre-AMC, University of Amsterdam, THE NETHERLANDS); Lopez MA, Secanell M, Sunol R and Vallejo P (Avedis Donabedian University Institute-Universitat Autónoma de Barcelona FAD. Red de investigación en servicios de salud en enfermedades crónicas REDISSEC, SPAIN); Bartels P (Central Denmark Region & The Department of Clinical Medicine, Aalborg University, Aalborg, DENMARK); Kristensen S (Central Denmark Region & Center for Healthcare Improvements, Aalborg University, Aalborg, DENMARK); Michel P and Saillour-Glenisson F (Comité de la Coordination de l'Evaluation Clinique et de la Qualité en Aquitaine, FRANCE) ; Vlcek F (Czech Accreditation Committee, CZECH REPUBLIC); Car M, Jones S and Klaus E (Dr Foster Intelligence-DFI, UK); Bottaro S and Garel P (European Hospital and Healthcare Federation-HOPE, BELGIUM); Saluvan M (Hacettepe University, TURKEY); Bruneau C and Depaigne-Loth A (Haute Autorité de la Santé-HAS, FRANCE); Shaw C (University of New South Wales, Australia); Hammer A, Ommen O and Pfaff H (Institute for Medical Sociology, Health Services Research and Rehabilitation Science, University of Cologne-IMVR, GERMANY); Groene O (London School of Hygiene and Tropical Medicine, UK); Botje D and Wagner C (The Netherlands Institute for Health Services Research-NIVEL, the NETHERLANDS); Kutaj-Wasikowska H and Kutryba B (Polish Society for Quality Promotion in Health Care-TPJ, POLAND); Escoval A and Lívio A (Portuguese Association for Hospital Development-APDH, PORTUGAL); Eiras M, Franca M and Leite I (Portuguese Society for Quality in Health Care-SPQS, PORTUGAL); Almeman F, Kus H and Ozturk K (Turkish Society for Quality Improvement in Healthcare-SKID, TURKEY); Mannion R (University of Birmingham, UK); Arah OA, DerSarkissian M, Thompson CA (Palo Alto Medical Foundation Research Institute, Palo Alto, California, USA); Wang A (University of California, Los Angeles-UCLA, USA); Thompson A (University of Edinburgh, UK).

## Appendix 2

(Tables A1 and A2).

**Table A1:** Patient involvement in planning for quality at hospital and departmental levels

Patients are involved in	Hospital quality manager		Head of pathway								*P*-value*
			AMI		Deliveries		Hip fracture		Stroke		
	*N*	%	*N*	%	*N*	%	*N*	%	*N*	%	
Total respondents, *N* (row %)	72	100	64	24.4	65	24.8	65	24.8	68	25.9	
Development of quality criteria											
Never	42	56.7	22	34.3	37	56.9	31	47.6	38	55.8	0.211
Sometimes	20	27.0)	28	43.7	17	26.1	19	29.2	21	30.8	
Usually	8	10.8	4	6.2	4	6.1	8	12.3	7	10.2	
Always	2	2.7	4	6.2	6	9.2	3	4.6	2	2.9	
Missing	0	0	6	9.3	1	1.5	4	6.1			
Design/organization of processes											
Never	45	60.8	28	43.7	43	66.1	36	55.3	41	60.2	0.217
Sometimes	21	28.3	24	37.5	11	16.9	17	26.1	18	26.4	
Usually	3	4.0	5	7.8	6	9.2	8	12.3	8	11.7	
Always	2	2.7	2	3.1	4	6.1	1	1.5	1	1.4	
Missing	1	1.3	5	7.8	1	1.5	3	4.6			
Quality committees											
Never	48	64.8	30	46.8	37	56.9	44	67.6	41	60.2	0.276
Sometimes	13	17.5	16	25.0	16	24.6	10	15.3	15	22.0	
Usually	4	5.4	7	10.9	3	4.6	2	3.0	8	11.7	
Always	7	9.4	3	4.6	6	9.2	6	9.2	3	4.4	
Missing	0	0	8	12.5	3	4.6	3	4.6	1	1.4	
Quality improvement projects											
Never	37	50.0	23	35.9	37	56.9	35	53.8	35	51.4	0.202
Sometimes	20	27.0	23	35.9	16	24.6	19	29.2	20	29.4	
Usually	9	12.1	9	14.0	4	6.1	3	4.6	9	13.2	
Always	6	8.1	1	1.5	5	7.6	4	6.1	3	4.4	
Missing	0	0	8	12.5	3	4.6	4	6.1	1	1.4	
Discussion of quality improvement project results											
Never	44	59.4	25	39.0	39	60.0	37	56.9	36	52.9	0.322
Sometimes	16	21.6	18	28.1	14	21.5	18	27.6	20	29.4	
Usually	5	6.7	10	15.6	4	6.1	5	7.6	10	14.7	
Always	7	9.4	1	1.5	4	6.1	2	3.0	1	1.4	
Missing	0	0	10	15.6	4	6.1	3	4.6	1	1.4	

**P*-value for differences in items across pathways from Fisher's exact test.

**Table A2:** Patient-centred care strategies in four departments

Patient-centred care strategies	All conditions		AMI		Deliveries		Hip fracture		Stroke		*P*-value
	*N*	%	*N*	%	*N*	%	*N*	%	*N*	%	
Total *N* (row %)	295	100	74	25.0	73	24.7	74	25.0	74	25.0	
Formal survey seeking views of patients and carers											
No or negligible compliance	43	14.5	12	16.2	9	12.3	11	14.8	11	14.8	0.952*
Low compliance	21	7.1	8	10.8	3	4.1	5	6.7	5	6.7	
Medium compliance	38	12.8	9	12.1	9	12.3	11	14.8	9	12.1	
High, extensive compliance	25	8.4	5	6.7	6	8.2	5	6.7	9	12.1	
Full compliance	165	55.9	38	51.3	45	61.6	42	56.7	40	54.0	
Missing	3	1.0	2	2.7	1	1.3					
Written policies on patients' rights											
No or negligible compliance	14	4.7	4	5.4	2	2.7	4	5.4	4	5.4	0.969*
Low compliance	12	4.0	2	2.7	2	2.7	5	6.7	3	4.0	
Medium compliance	33	11.1	10	13.5	6	8.2	9	12.1	8	10.8	
High, extensive compliance	23	7.7	5	6.7	5	6.8	6	8.1	7	9.4	
Full compliance	210	71.1	51	68.9	57	78.0	50	67.5	52	70.2	
Missing	3	1.0	2	2.7	1	1.3					
Patient information literature includes guidelines											
No or negligible compliance	70	23.7	14	18.9	11	15.0	24	32.4	21	28.3	0.054**
Low compliance	32	10.8	10	13.5	5	6.8	8	10.8	9	12.1	
Medium compliance	40	13.5	13	17.5	7	9.5	14	18.9	6	8.1	
High, extensive compliance	39	13.2	8	10.8	14	19.1	5	6.7	12	16.2	
Full compliance	111	37.6	27	36.4	35	47.9	23	31.0	26	35.1	
Missing	3	1.0	2	2.7	1	1.3					
Fact sheets for post-discharge care											
No or negligible compliance	46	15.5	7	9.4	20	27.3	11	14.8	8	10.8	0.033**
Low compliance	29	9.8	6	8.1	3	4.1	9	12.1	11	14.8	
Medium compliance	29	9.8	7	9.4	4	5.4	12	16.2	6	8.1	
High, extensive compliance	60	20.3	19	25.6	16	21.9	13	17.5	12	16.2	
Full compliance	128	43.3	33	44.5	29	39.7	29	39.1	37	50.0	
Missing	3	1.0	2	2.7	1	1.3					

**P*-value for differences in items across pathways from Fisher's exact test.

***P*-value for differences in items across pathways from χ^2^ test.
